# Fluoride in drinking water and fluorosis in an Argentine province: a spatial and epidemiologic analysis

**DOI:** 10.3389/froh.2026.1843215

**Published:** 2026-06-08

**Authors:** E. B. Gonzalez, L. A. R. Valadas, C. L. Leyes, M. Carabajal, M. M. Barros Uriburu, A. F. Squassi, A. L. Sorazabal

**Affiliations:** 1 Universidad de Buenos Aires, Facultad de Odontología, Cátedra de Odontología Preventiva y Comunitaria, Buenos Aires, Argentina; 2 Universidad de Buenos Aires, Facultad de Odontología, Instituto de Investigaciones en Salud Publica, Buenos Aires, Argentina; 3 Universidad de Buenos Aires, Facultad de Odontología, Cátedra de Preclinica de Rehabilitación Protética, Buenos Aires, Argentina

**Keywords:** Argentina, dental fluorosis, fluoride exposure, fluoride in drinking water, groundwater, spatial analysis

## Abstract

**Introduction:**

Excessive fluoride exposure during enamel development is a major risk factor for dental fluorosis, particularly in populations relying on groundwater sources with naturally elevated fluoride concentrations. Rural regions with limited access to centralized water systems may present heterogeneous exposure patterns influenced by environmental and socioeconomic factors.

**Aim:**

This study aimed to evaluate fluoride concentration in drinking water and the prevalence of dental fluorosis in schoolchildren from La Rioja, Argentina.

**Method:**

A cross-sectional ecological study was conducted including 469 schoolchildren aged 6–12 years from San Blas de los Sauces and Arauco. Only children born and continuously residing in the study area were included. Dental fluorosis was assessed using the Thylstrup and Fejerskov (T-F) index by calibrated examiners (Kappa > 0.8). Fluoride concentration was determined in 82 georeferenced groundwater samples using an ion-selective electrode. Spatial analysis included Moran's I and Local Indicators of Spatial Association (LISA). Associations between fluorosis severity and locality were evaluated using chi-square tests with Monte Carlo simulation.

**Results:**

The overall prevalence of dental fluorosis was 46.3% (95% CI: 40.8–49.9), with moderate fluorosis as the most frequent category. A significant association between locality and fluorosis severity was observed (*p* < 0.0001; Cramér's V = 0.23), with marked spatial heterogeneity. Fluoride concentrations varied across localities, with several exceeding the 1.5 mg/L threshold. Spatial analysis revealed significant clustering of high fluoride concentrations (Moran's I = 0.16; *p* = 0.006) and localized hotspots.

**Discussion:**

Although areas with higher fluoride concentrations and higher fluorosis severity were spatial overlapped, this was therefore interpreted as a descriptive pattern of co-occurrence, not as evidence of causality. A high prevalence of dental fluorosis with significant spatial variability was identified in the study area. The absence of a direct association between fluoride levels and fluorosis severity suggests a complex exposure pattern influenced by multiple factors.

**Conclusion:**

The integration of epidemiological, environmental, and spatial data provides a useful framework for identifying high-risk areas and supporting targeted public health interventions in regions with decentralized water systems.

## 1 Introduction

Fluoride is one of the most electronegative elements and is commonly found in nature in its ionic form (1). As a collective strategy for the prevention of dental caries, water fluoridation has been widely implemented worldwide and remains one of the most effective public health measures at the population level (2, 3). However, its beneficial effects depend on dose, timing, and patterns of exposure. Excessive fluoride intake during enamel development is a major risk factor for dental fluorosis (4). This condition may present as enamel opacities ranging from mild discoloration to severe structural damage (3, 5), particularly in children exposed to elevated fluoride levels from early life. In socioeconomically and geographically vulnerable regions, access to safe drinking water is often limited, and reliance on groundwater sources such as wells remains common (6, 7). In these settings, fluoride concentrations may vary significantly depending on geological and environmental conditions, frequently exceeding recommended levels and increasing the risk of chronic exposure (8, 9).

Fluoride levels in groundwater are influenced by multiple factors, including the geological composition of soils and rocks, hydrogeochemical processes, and climatic conditions. The dissolution of fluoride-bearing minerals, along with rainfall patterns, infiltration rates, and aquifer exploitation, contributes to variability in fluoride concentrations. In addition, anthropogenic activities may affect water quality through the introduction of contaminants such as heavy metals, nitrates, and pesticides (8, 10).

Mountainous regions play a key role in determining water chemistry due to water–rock interactions. In La Rioja, Argentina, this process is strongly influenced by the Sierra de Velasco, a crystalline basement uplift composed predominantly of granitic and gneissic units (11, 12). Fluoride-bearing minerals, such as biotite and fluorapatite, have been identified as primary geogenic sources of fluoride in groundwater systems (12). Their dissolution during subsurface flow, combined with geomorphological features such as elongated valleys and alluvial fans, promotes prolonged water–rock contact and contributes to elevated fluoride concentrations (13).

The prevalence of dental fluorosis has been widely documented in populations exposed to groundwater with high fluoride levels (8–10, 14), particularly when concentration exceeds 1.5 mg/L the upper limit recommended by the World Health Organization (5, 9).

Dental fluorosis is commonly assessed using standardized indices. Among these, the Thylstrup and Fejerskov index (T-F index) is widely used due to its sensitivity in detecting subtle enamel changes and its ability to reflect the biological gradient of fluoride exposure (15).

Despite the known geogenic conditions in La Rioja, there is a lack of updated epidemiological data for specific departments such as San Blas de los Sauces and Arauco. Although regional fluoride levels are known to be high, the prevalence and severity of dental fluorosis in these pediatric populations have not been systematically evaluated.

Therefore, this study aimed to evaluate the fluoride concentration in drinking water, and the prevalence of dental fluorosis in schoolchildren from San Blas de los Sauces and Arauco, La Rioja, Argentina.

## 2 Materials and methods

### 2.1 Study area and population

**Figure 1:**
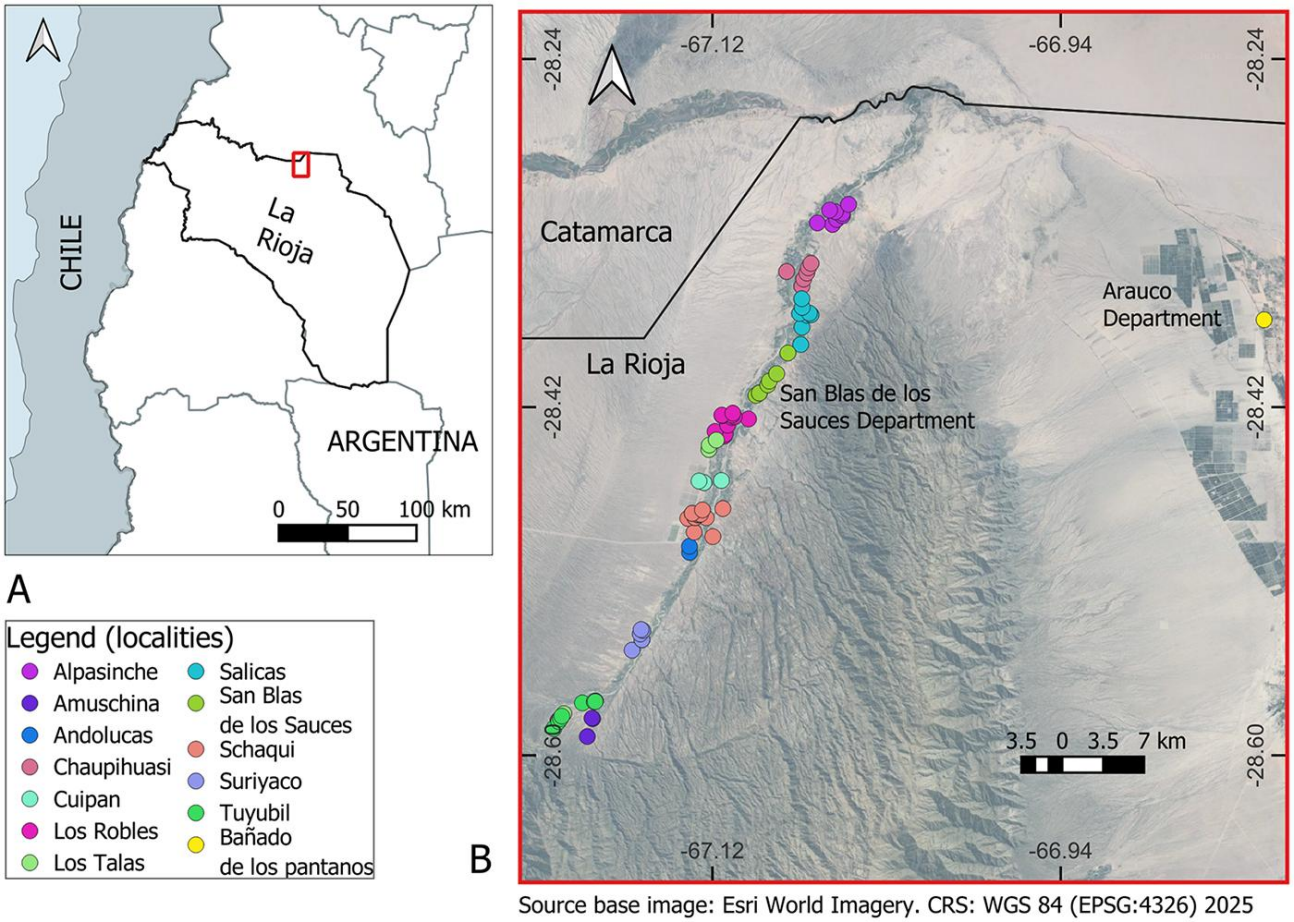
Geographic location of the study area and water sampling sites. **(A)** Regional context of La Rioja Province within Argentina. **(B)** Detailed view of the study area in the San Blas de los Sauces and Arauco departments, showing the spatial distribution of water sampling sites by locality. Colors in the legend correspond to each sampled locality. Background imagery: Esri World Imagery**.**

The study was conducted in the Department of San Blas de los Sauces, located in the northwestern region of La Rioja Province, Argentina (Figure 1). This area has a low population density (2.4 inhabitants/km^2^) and an estimated population of approximately 4,000 inhabitants distributed across multiple small rural localities aligned along the Los Sauces River valley (16). The department comprises 12 localities situated along the Los Sauces River and longitudinally connected by National Route 40: Alpasinche, Chaupihuasi, Salicas, San Blas de los Sauces, Los Robles, Las Talas, Cuipán, Schaqui, Andolucas, Suriyaco, Amuschina, and Tuyubil. The primary source of drinking water in these communities is groundwater obtained from private and public wells. The local economy is mainly based on agricultural production, including raisins, olives, pistachios, and walnuts (16).

The locality of Bañado de los Pantanos, located in the Arauco Department to the east of San Blas de los Sauces, was also included in the study. This rural locality has a population of 353 inhabitants distributed over 19.26 km^2^. Drinking water is supplied from a single groundwater well, stored in a central tank, and distributed to households. The local economy is primarily based on agriculture, particularly the cultivation of jojoba and cumin (16).

The study population consisted of primary school children examined within a pediatric dentistry outreach program conducted across nine schools in the department between 2018 and 2022. Only children who were born and continuously resided in the study area were included, in order to ensure consistent exposure to local water sources during the critical period of enamel development and to minimize exposure misclassification.

Children with a history of tetracycline use, incomplete records, lack of informed consent, or duplicate entries were excluded. When multiple records existed for a participant, the most recent examination was retained. The final analytical sample comprised 469 children.

### 2.2 Fluorosis classification and clinical examination

Each child underwent a clinical examination to assess dental fluorosisusing the Thylstrup and Fejerskov (T-F) index (15), performed under standardized conditions, using light, dental mirror and WHO periodontal probe. The dental exams were performed by 3 calibrated researchers (M.M.B.U., M.C., C.L.), under the supervision of a reference examiner (A.L.S).The calibration strategy consisted of:(a) Expository class (2 h) with photographs (*n* = 36) aimed at the recognition of the categories established in the T-F Index and the cut-off points between the different categories and the protocol to carry out the diagnosis. (b) Clinical practice (20 h), which included the following phases: I. Assignment to each operator of 6 volunteer subjects with fluorosis with different grades of severity. II. Observation and recording of the findings in an *ad hoc* spread sheet, in charge of each independent operator. III. Re-evaluation of each patient by the reference examiner and recording of the findings.

Inter- and intra-examiner reliability were assessed using Cohen's kappa statistic, achieving values greater than 0.80, indicating substantial agreement. During fieldwork, each child was examined independently. In cases of disagreement, a consensus diagnosis was established through joint evaluation; when consensus was not reached, the reference examiner's classification was adopted as the final diagnosis.

Participants were classified into four hierarchical based on Thylstrup and Fejerskov (T-F) index (15). A fluorosis-free (sound) status was assigned only when all examined surfaces scored 0. When fluorosis was present, the diagnosis was defined according to the highest degree of severity observed: mild for individuals with at least one surface scored 1 or 2; moderate for surfaces with values of 3 or 4; and severe for cases with at least one surface scored 5 or higher.

### 2.3 Water sampling and fluoride determination

Fluoride concentrations in drinking water were determined through two sampling campaigns conducted in 2019 and 2022, yielding a total of 82 georeferenced groundwater samples (Table 1).

**Table 1:** Number of water samples per locality.

Locality	Water samples
Alpansinche	10
Amuschina	4
Andolucas	2
Bañado de los pantanos	2
Chaupihuasi	6
Cuipán	3
Los Robles	11
Los Talas	4
Salicas	8
San Blas	7
Schaqui	13
Suriyaco	6
Tuyubil	7

Samples were collected from the primary water sources used for human consumption in each locality, predominantly groundwater from wells (Figure 1). Geographic coordinates were recorded using a Garmin® eTrex GPS device.

Fluoride concentration was measured using potentiometry with a combined ion-selective electrode (HI 4110, Hanna Instruments®), following established protocols (2). Each sample was analyzed in duplicate. Prior to measurement, samples were buffered with TISAB II solution and adjusted with NaOH as required. Calibration curves were constructed using standard fluoride solutions ranging from 0.5 to 32.0 μg/mL, achieving coefficients of determination (R^2^) greater than 0.999. Fluoride concentrations were expressed in parts per million (ppm).

### 2.4 Statistical analysis

This study was designed as a cross-sectional ecological analysis, in which individual clinical outcomes were evaluated alongside environmental exposure data measured at the locality level. Given this design, results were interpreted at the group level, and no causal inferences at the individual level were made.

Descriptive and inferential analyses were conducted using R software (version 4.2.0; R Core Team, Vienna, Austria), with the packages *dplyr*, *ggplot2*, *tidyverse*, and *ggpattern*. ChatGPT (GPT-5.5, OpenAI, San Francisco, CA, USA) was used to assist in the development and review of R code.

An exploratory data analysis was performed to characterize the study population and describe the distribution of dental fluorosis according to age, sex, and locality. Prevalence was calculated as the proportion of affected individuals within the total sample, stratified by severity category. Ninety-five percent confidence intervals (95% CI) were estimated using exact binomial methods.

Associations between fluorosis severity and locality were evaluated using Pearson's chi-square test. When expected cell counts were below five, significance was assessed using Monte Carlo simulation (10,000 replicates). The strength of associations was quantified using Cramer's V. Adjusted standardized residuals were calculated to identify cells contributing to significant associations, with values exceeding ±1.96 considered statistically relevant.

### 2.5 Spatial analysis

Spatial analysis was conducted to evaluate the geographic distribution of fluoride concentrations. Global spatial autocorrelation was assessed using Moran's I statistic with 999 Monte Carlo permutations. A spatial weights matrix was constructed using a k-nearest neighbors approach (k = 10), with row-standardized weights and projected coordinates (UTM) to ensure accurate distance calculations.

Directional variograms (0°, 45°, 90°, and 135°) were examined to assess spatial anisotropy. Due to the elongated configuration of the study area and irregular sampling distribution, stable variogram models could not be fitted, and therefore kriging interpolation methods were not applied. Inverse Distance Weighting (IDW) interpolation was also evaluated but excluded due to the generation of spatial artifacts.

To complement the global analysis, Local Indicators of Spatial Association (LISA) were computed to identify localized clusters and spatial outliers. This approach allowed for a more detailed characterization of spatial patterns, acknowledging the overall variability in fluoride distribution.

Differences in fluoride concentrations between localities were assessed using the Kruskal–Wallis test, followed by pairwise Wilcoxon rank-sum tests with Holm correction for multiple comparisons. Effect size was estimated using epsilon-squared (*ε*^2^).

## 3 Results

### 3.1 Sample characterization and global prevalence

**Figure 2:**
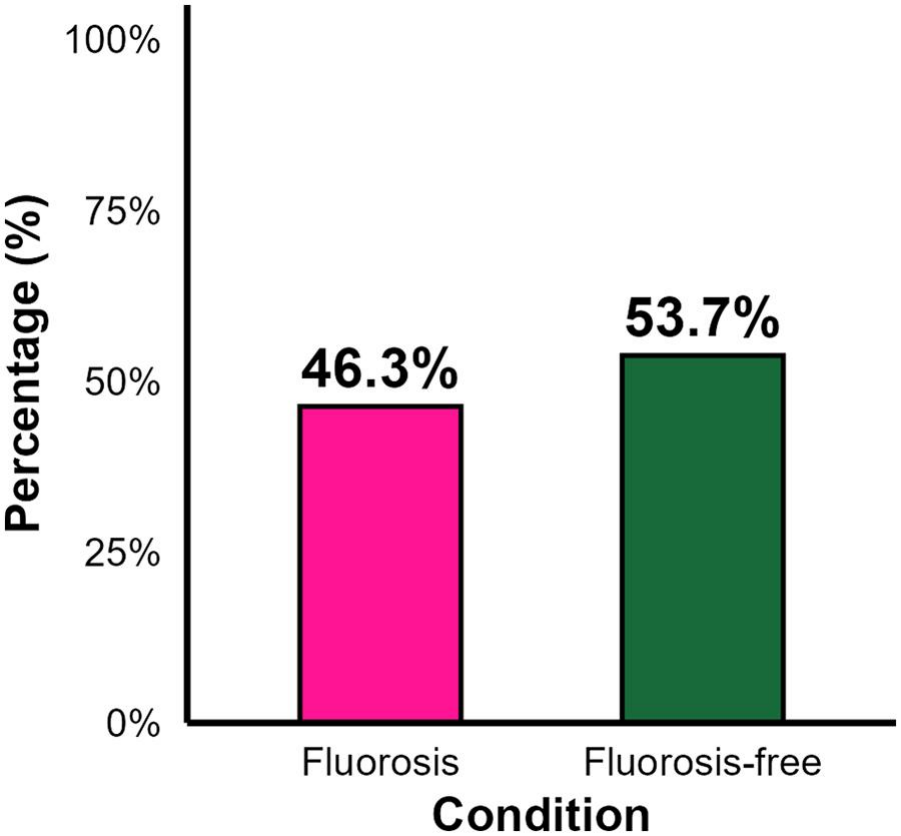
Global prevalence of dental fluorosis and fluorosis-free status in the study population (*n* = 469).

A total of 469 schoolchildren were included in the final analysis. Overall, 46.3% (95% CI: 40.8–49.9) met diagnostic criteria for dental fluorosis, while 53.7% were classified as fluorosis-free (Figure 2).

**Figure 3:**
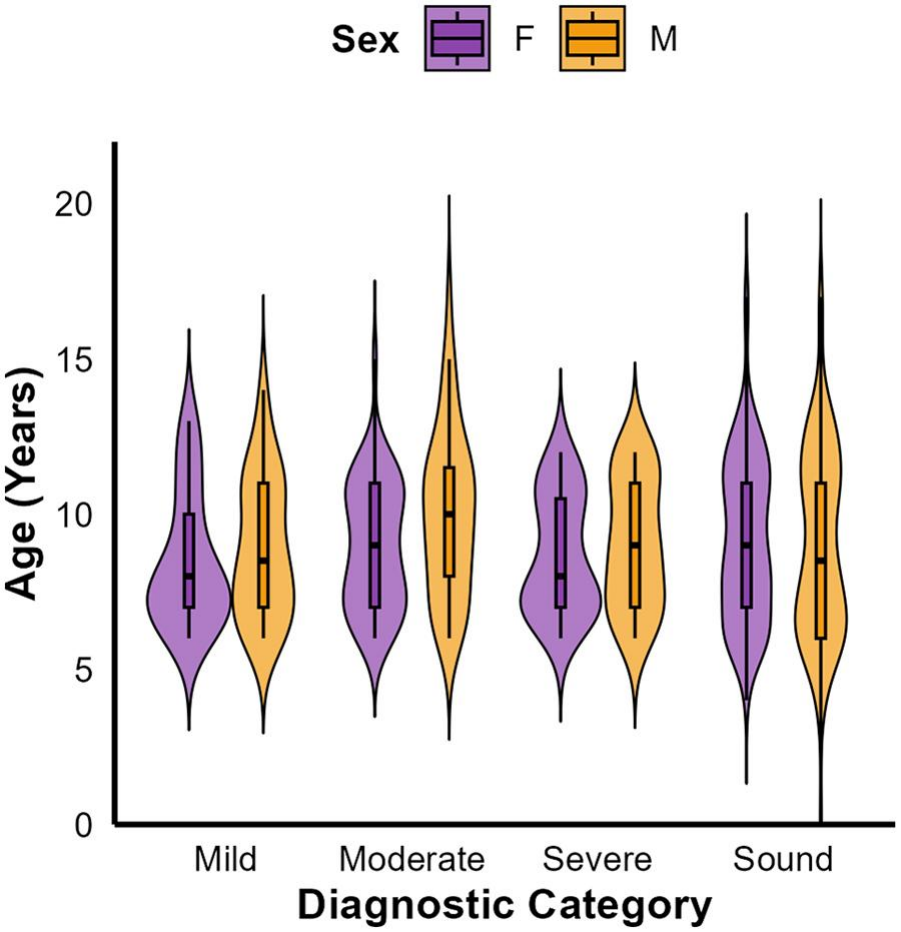
Age distribution by diagnostic category and sex. Violin plots show the kernel density estimation; internal boxplots represent the median and interquartile ranges. F, Female; M, Male.

Among affected individuals, moderate fluorosis was the most frequent clinical presentation (46.5%), followed by mild (28.1%) and severe forms (25.3%) (Table 2), indicating that a substantial proportion of cases presented clinically evident enamel alterations. Age distribution was consistent across all diagnostic categories and sexes, with a median of approximately 9 years (Figure 3). No significant differences in age distribution were observed between males and females or across fluorosis severity categories.

**Table 2:** Distribution of dental fluorosis severity categories among affected individuals.

Severity level	Total cases	Percentage
Mild	68	28.1
Moderate	101	46.5
Severe	55	25.3

### 3.2 Spatial distribution of fluorosis severity by locality

**Figure 4:**
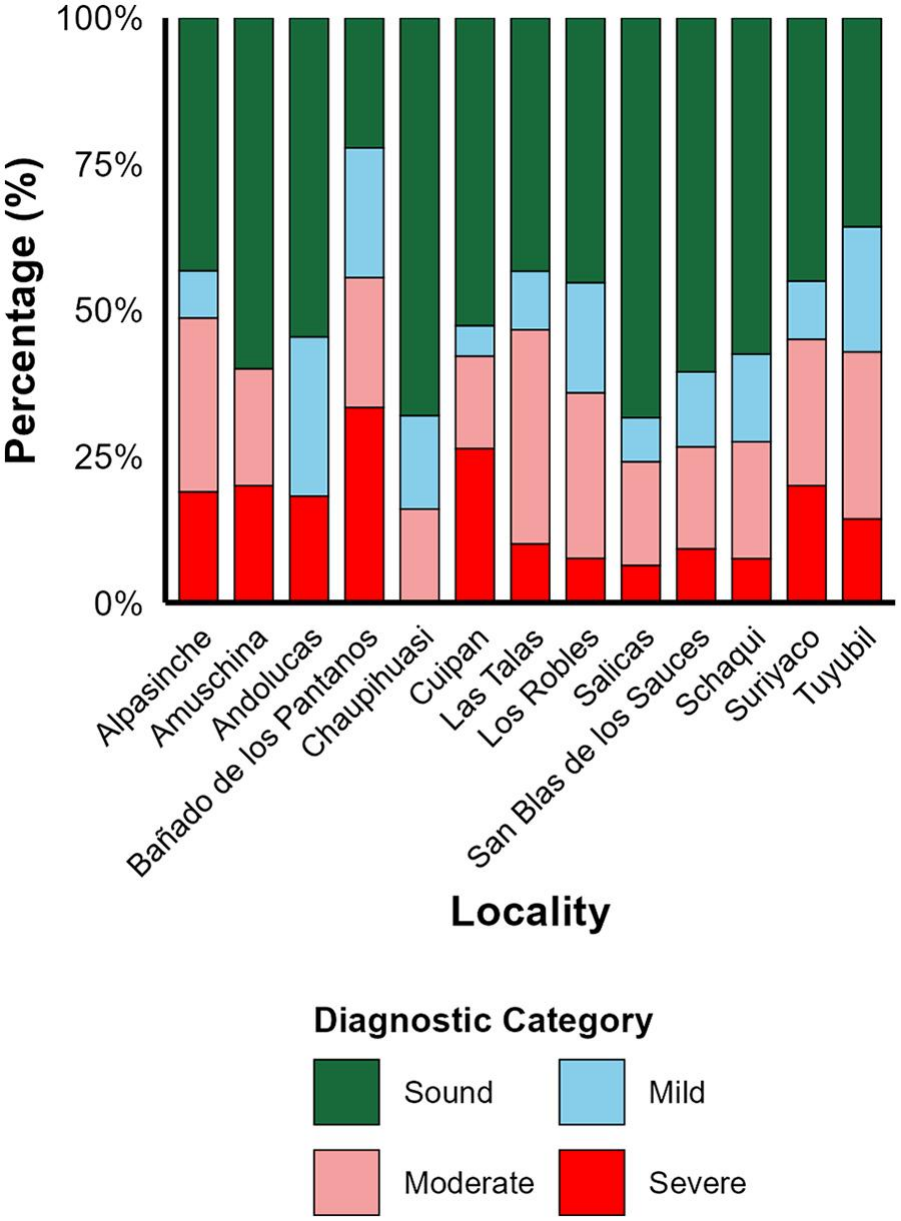
Relative frequency of fluorosis diagnostic categories across the studied localities. The stacked bars represent the proportional distribution of each category (Sound, Mild, Moderate, and Severe) within each geographical site.

The distribution of fluorosis severity varied across localities (Figure 4). While most areas showed a predominance of fluorosis-free individuals, the proportion and severity of affected cases differed between sites.

Bañado de los Pantanos presented the highest proportion of severe fluorosis, being the only site where this category predominated. Cuipan and Suriyaco showed elevated proportions of moderate and severe cases. In contrast, Salicas and San Blas de los Sauces exhibited higher proportions of fluorosis-free individuals. Other localities, such as Alpasinche and Los Robles, displayed intermediate distributions.

### 3.3 Locality–fluorosis association analysis

A Pearson chi-square test with Monte Carlo simulation (10,000 replicates) showed a statistically significant association between locality of residence and fluorosis severity (*p* < 0.0001), with a moderate effect size (Cramér's V = 0.23).

**Figure 5:**
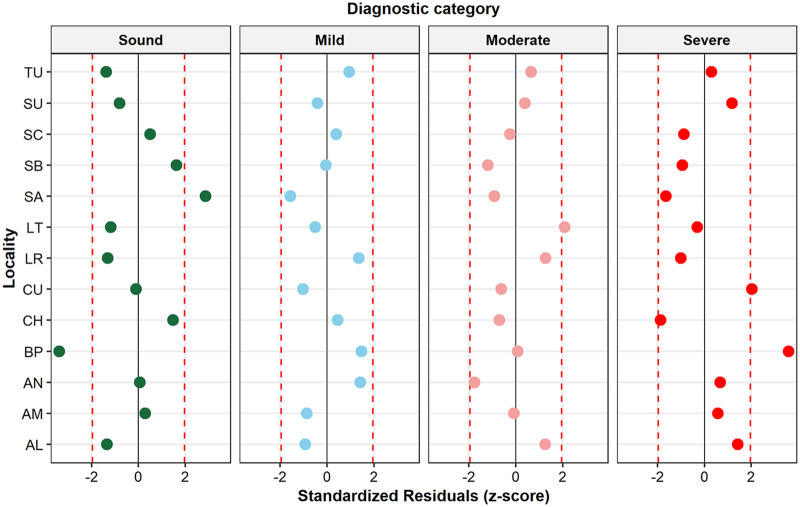
Association between fluorosis severity and locality of residence. Dot plot of standardized residuals **(z)** from the Pearson chi-square test across diagnostic categories. Dashed lines indicate significance thresholds (|z| = 1.96). Positive values indicate excess cases and negative values indicate deficits. Localities: AL, Alpasinche; AM, Amuschina; AN, Andolucas; BP, Bañado de los Pantanos; CH, Chaupihuasi; CU, Cuipan; LR, Los Robles; LT, Las Talas; SA, Salicas; SB, San Blas de los Sauces; SC, Schaqui; SU, Suriyaco; TU, Tuyubil.

Post-hoc analysis based on standardized residuals (Figure 5) identified the specific locality–severity combinations contributing to this association. Bañado de los Pantanos (BP) showed an excess of severe cases (z = 3.58) and a deficit of fluorosis-free individuals (z = −3.37).

Cuipan (CU) also exhibited a significant excess of severe fluorosis (z = 2.01), while Las Talas (LT) showed an excess of moderate cases (z = 2.07). In contrast, Salicas (SA) presented an excess of fluorosis free individuals (z = 2.87).

### 3.4 Spatial fluoride concentration in water sources

**Figure 6:**
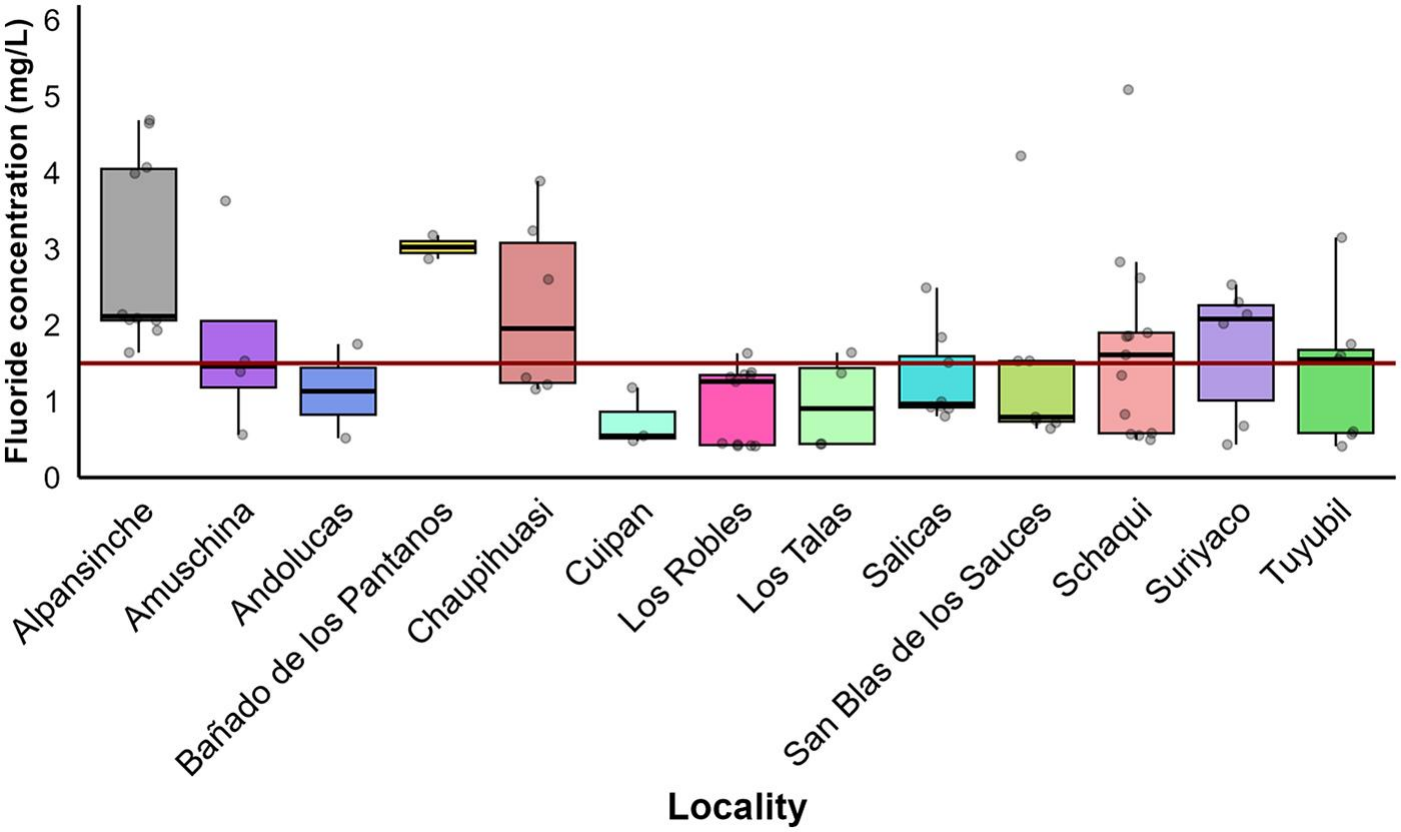
Fluoride concentration by locality**.** Boxplots representing the distribution of fluoride levels (mg/L) across the sampled localities. Each box indicates the interquartile range (IQR), and the horizontal line within the box represents the median. Individual data points are displayed to show sample dispersion. The dashed red line indicates the maximum permissible limit for fluoride in drinking water according to the World Health Organization (WHO; 1.5 mg/L).

Fluoride concentrations varied across localities (Figure 6). Several sites showed values exceeding the recommended threshold of 1.5 mg/L established by the WHO and Argentine Food Code (CAA) (17).

Alpasinche and Bañado de los Pantanos exhibited the highest concentrations, with most samples above this threshold. Chaupihuasi also presented elevated levels. In contrast, Cuipan and Los Robles consistently remained below the recommended limit.

**Figure 7:**
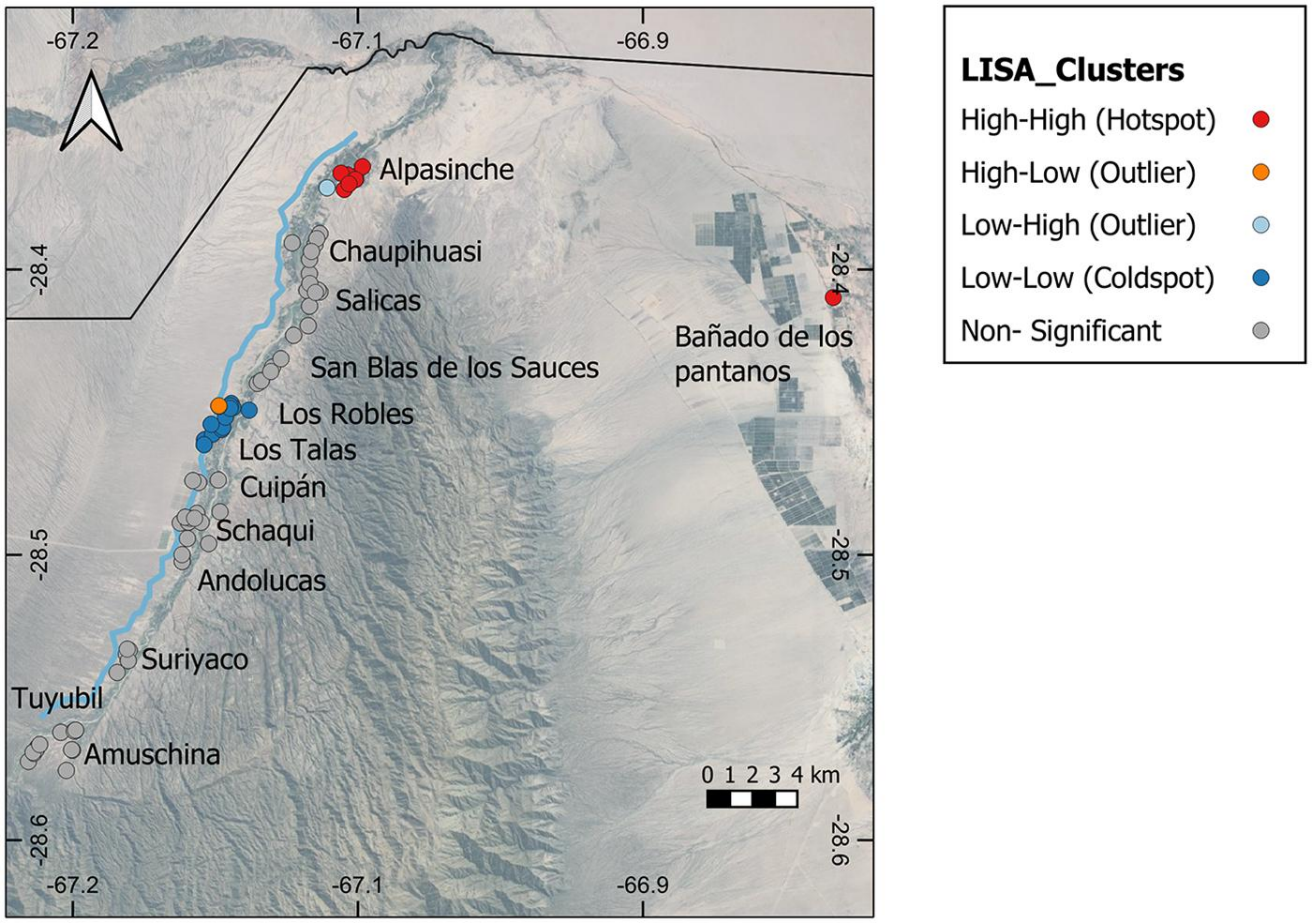
Local indicators of spatial association (LISA) for water fluoride concentrations. Significant High-High hotspots (*p* < 0.05, red circles) are localized in the northern sector (Alpasinche and Chaupihuasi) and at Bañado de los Pantanos. Significant Low-Low coldspots (blue circles) were identified in the central-western sector around Los Robles. Grey symbols represent non-significant locations (*p* > 0.05). Background Imagery: Esri World Imagery. CRS: UTM Zone 19S (EPSG:32719). 2025.

Global spatial autocorrelation analysis (Moran’s I = 0.16; *p* = 0.006) indicated a weak but statistically significant positive spatial pattern. Local Indicators of Spatial Association (LISA) identified two High–High clusters (hotspots): one in the northern sector (Alpasinche and Chaupihuasi) and another in Bañado de los Pantanos (Figure 7). A Low–Low cluster was observed in the central-western sector.

When considered alongside fluorosis distribution patterns, some geographic overlap between higher fluoride concentrations and higher fluorosis severity was observed. However, due to sampling heterogeneity, limited sample sizes in some localities, and the absence of longitudinal exposure data, no formal statistical association between environmental fluoride levels and clinical outcomes was established.

A Kruskal–Wallis test confirmed significant differences in water fluoride concentrations across localities (H = 32.218, df = 13, *p* = 0.002), with a large effect size (*ε*^2^ = 0.387). Pairwise *post-hoc* comparisons with Holm correction identified a statistically significant difference specifically between Alpasinche and Los Robles (*p* < 0.001).

## 4 Discussion

The present study identified a dental fluorosis prevalence of 46.3% among schoolchildren, with a predominance of moderate severity. These findings are consistent with studies conducted in populations exposed to naturally fluoridated groundwater, where fluorosis prevalence increases when fluoride concentrations exceed recommended thresholds, particularly in rural and environmentally vulnerable settings (8, 9). Notably, similar prevalence values have been reported in children aged 6–12 years living in endemic areas, supporting the external validity of the present results.

The predominance of moderate fluorosis observed in this population aligns with previous reports from regions with chronic exposure to intermediate-to-high fluoride concentrations. In such contexts, sustained exposure during enamel development typically results in clinically evident alterations without necessarily progressing to severe structural damage (5, 18).

The study population was concentrated within the 6–12-year age range, with a median age of 9 years, corresponding to the period of permanent tooth eruption. This age group is commonly used in epidemiological studies, as fluorosis become clinically detectable after tooth eruption and reflects fluoride exposure during earlier developmental stages (8, 9).

A central finding of this study is the marked spatial heterogeneity in fluorosis prevalence and severity across localities. This variability is consistent with the known heterogeneity of fluoride concentrations in groundwater, which are influenced by geological and hydrochemical factors; however, given the ecological design, these patterns should be interpreted as co-occurrence rather than evidence of direct association. The spatial clustering identified in this study further supports the presence of geographically structured exposure patterns.

Within this regional context, the Sierra de Velasco represents a major geogenic source of fluoride, where the dissolution of fluoride-bearing minerals contributes to groundwater enrichment (12, 13). However, at the local scale, fluoride concentrations were not uniformly distributed. Some localities exhibited low concentrations despite sharing the same geological background, suggesting that local hydrogeochemical processes, including groundwater flow dynamics and mineral interactions, play an important role in modulating fluoride availability. Similar mechanisms have been described in other regions, where fluoride distribution depends on localized geochemical conditions rather than solely on lithology (19, 20).

Such heterogeneity may also reflect differences in well depth, groundwater extraction methods, and the potential mixing of aquifers with distinct hydrochemical characteristics, which can result in non-uniform fluoride distributions even within a shared geological framework.

Although several localities presented fluoride concentrations exceeding the Recommended threshold of 1.5 mg/L, no direct statistical association was observed between fluoride levels and fluorosis severity, reinforcing that the present analysis reflects partial environmental exposure rather than individual-level dose.

In addition to drinking water, total fluoride intake is influenced by dietary sources, processed beverages and fluoride-containing dental products (2). Furthermore, individual variability in water consumption and potential mobility between localities may attenuate ecological associations.

Accurate quantification of individual fluoride exposure requires the integration of multiple variables beyond water concentration alone, including ingestion rate, exposure frequency, duration of residence, and body weight, none of which were available at the individual level in the present study. This limitation is particularly inherent to research conducted in small, geographically isolated rural areas where baseline epidemiological and environmental data are largely absent. Notably, the study population presents minimal residential mobility (16), and groundwater from local wells constitutes the primary and in most localities the only source of drinking water, which supports the use of water fluoride concentration as a meaningful, albeit partial, indicator of chronic fluoride exposure in this specific context.

Therefore, the present findings should be interpreted as reflecting a partial assessment of fluoride exposure, primarily based on drinking water sources.

Another important limitation relates to the temporal mismatch between exposure and outcome. Dental fluorosis reflects fluoride intake during enamel formation, which occurs years before clinical examination. Therefore, current measurements of fluoride concentration may not accurately represent historical exposure conditions (10).

Additionally, fluoride exposure was assessed at the locality level, whereas fluorosis was evaluated at the individual level. This results should be interpreted within an ecological framework, avoiding individual-level causal inferences and recognizing the limitations of aggregated exposure assessment. Although areas with higher fluoride concentrations and higher fluorosis severity were spatial overlapped, this was therefore interpreted as a descriptive pattern of co-occurrence, not as evidence of causality.

From a broader health perspective, dental fluorosis is a recognized marker of excessive fluoride exposure, and high levels of chronic intake may also be associated with systemic effects such as skeletal fluorosis in severely exposed populations (21). In groundwater-dependent regions, the presence of other environmental elements, including heavy metals or naturally occurring compounds, may further contribute to the overall exposure profile (21, 22).

The identification of localities with higher fluorosis burden highlights the importance of place-based approaches in public health. Environmental conditions, water access, and socioeconomic factors may interact to produce heterogeneous exposure patterns within the same region (7, 14). In this context, dental fluorosis can be considered not only a clinical condition but also an indicator of environmental exposure and social vulnerability.

### 4.1 This study has several limitations

The cross-sectional design and the temporal mismatch between fluoride exposure and clinical outcomes limit causal interpretation. Water sampling was not uniform across localities, and the number of samples was limited in some areas, which may affect comparability. In addition, individual-level exposure data and other fluoride sources were not assessed. Spatial analysis was exploratory and did not include uncertainty quantification. Future studies incorporating longitudinal designs and comprehensive assessment of total fluoride intake are warranted.

Despite these limitations, this study provides an integrated approach combining epidemiological data, environmental measurements, and spatial analysis. The use of georeferenced sampling and spatial statistics allowed the identification of geographic patterns that may not be detected through conventional analyses. This approach may represent a useful tool for surveillance and risk assessment in regions with decentralized water systems.

## 5 Conclusion

This study identified a high prevalence of dental fluorosis in children from La Rioja, Argentina, with marked spatial variability in both fluoride concentration in drinking water and disease distribution. The absence of a direct association between water fluoride levels and fluorosis severity suggest the complexity of fluoride exposure likely influenced by multiple sources and contextual factors beyond drinking water alone.

The integration of epidemiological data with environmental measurements and spatial analysis proved to be a valuable approach for identifying high-risk areas and understanding exposure patterns. This framework may contribute to improving surveillance strategies in regions with decentralized water systems.

From a public health perspective, these findings underscore the need for localized monitoring of fluoride levels and the implementation of targeted interventions aimed at reducing exposure in vulnerable populations.

## Data Availability

The raw data supporting the conclusions of this article will be made available by the authors, without undue reservation.
